# Noninvasive Identification of Immune-Related Biomarkers in Hepatocellular Carcinoma

**DOI:** 10.1155/2019/2531932

**Published:** 2019-08-18

**Authors:** Ling Li, Huijia Zhao, Binyao Chen, Kang Huang, Zhuowen Hao, Zhipeng Fan, Gongpeng Sun, Jianguo Wu, Ning Li, Qifa Ye, Jiang Yue

**Affiliations:** ^1^Department of Pharmacology, Basic Medical School of Wuhan University, Wuhan 430071, China; ^2^Zhongnan Hospital of Wuhan University, Institute of Hepatobiliary Diseases of Wuhan University, Transplant Center of Wuhan University, Hubei Key Laboratory of Medical Technology on Transplantation, Wuhan, Hubei 430071, China; ^3^College of Life Sciences of Wuhan University, State Key Laboratory of Virology, Wuhan, Hubei 430071, China; ^4^Department of Cardiology, Renmin Hospital of Wuhan University, Cardiovascular Research Institute of Wuhan University, Hubei Key Laboratory of Cardiology, Wuhan, China; ^5^The 3rd Xiangya Hospital of Central South University, Research Center of National Health Ministry on Transplantation Medicine Engineering and Technology, Changsha 410013, China

## Abstract

Primary liver carcinoma is one of the most common malignant tumors with a poor prognosis. This study aims to uncover the potential mechanisms and identify core biomarkers of hepatocellular carcinoma (HCC). The HCC gene expression profile GSE49515 was chosen to analyze the differentially expressed genes from purified RNA of peripheral blood mononuclear cells, including 10 HCC patients and 10 normal individuals. GO and KEGG pathway analysis and PPI network were used, and the enrichment of the core genes out of 15 hub genes was evaluated using GEPIA and GSEA, respectively. We employed flow cytometry to count mononuclear cells to verify our opinions. In this analysis, 344 DEGs were captured, including 188 upregulated genes and 156 downregulated genes; besides that, 15 hub genes were identified. GO analysis and KEGG analysis showed the DEGs were particularly involved in immune response, antigen processing and presentation, and infection and inflammation. The PPI network uncovered 2 modules were also mainly involved in immune response. In conclusion, our analysis disclosed the immune subversion was the major signature of HCC associated closely with JUN, VEGFA, TNFSF10, and TLR4, which could be novel noninvasive biomarkers in peripheral blood and targets for early diagnosis and therapy of HCC.

## 1. Introduction

Hepatocellular carcinoma (HCC) is one of the most common malignancies, especially in the aged, which accounts for approximately 90% of all primary liver cancers severely threatening public health [1]. The mechanism of HCC is a complex process associated with the incremental accumulation of gene mutation, giving rise to abnormal immune subversion, cell cycle, and angiogenesis [2–4]. As for immune subversion, effector immune cells could execute immune control of HCC, which efficiently decrease malignant transformed cells. However, progression of HCC clearly certifies failure of tumor immune control suggesting inhibition of anticancer immune responses [5]. Especially, tumor-related mononuclear cells collaborate within an inflammatory network, which result in the immune privilege in the tumor environment [6]. Therefore, immunosuppressive mononuclear cells are equivalent to heterogeneous cell lines, including lymphocytes and monocytes cooperating by direct cell contact, secretion of cytokines, or production of extracellular matrix, which lead to the suppression of the immune response in the tumor milieu [7].

Currently, imageological examination and pathological biopsy are the conventional diagnostic methods of HCC [8]. However, imaging displays poor specificity, and pathological biopsy is an invasive method which may result in iatrogenic injury [9]. Therefore, serum biomarkers are routinely used for tumor diagnostic. For example, alpha-fetoprotein (AFP) has been widely used in clinical practice [10]. Although many studies have reported the accuracy of AFP for HCC, solely AFP still has some false-positive or false-negative rate [11]. Hence, the identification of specific and sensitive biomarkers is necessary in order to achieve accurate diagnosis and treatment of HCC as early as possible, especially noninvasive biomarkers.

High-throughput gene microarray is increasingly being widely used, which can analyze cancer and noncancer samples indicating us tumor-related genes at multiple levels from molecular diagnosis and pathological classification to therapeutic evaluation and prognosis prediction, as well as drug sensitivity and neoplasm recurrence [12–14]. However, the use of microarrys in clinical application is restricted by countless number of genes identified by gene profiling, lack of both repeatability and independent verification, and requirement for complex statistical analyses. Moreover, most of the microarrys are based on the genes in tissues which are difficult to detect except by invasive methods [15]. Therefore, in order to put these expression profiles into clinical applications as soon as possible, it is necessary to identify an appropriate amount of serum genes and develop a suitable way that can be done by routine assay.

In this study, we downloaded the HCC gene expression profile GSE49515 in the Gene Expression Omnibus (GEO, http://www.ncbi.nlm.nih.gov/geo/), an online public collection database for microarray data and used GEO2R online software to compare gene expression profiles of tumor cells with normal liver cells to identify differentially expressed genes (DEGs). Then, we constructed the protein-protein interaction (PPI) network of the DEGs and selected 15 hub genes according to a high degree of connectivity. Following this, we analyzed gene ontology (GO) and pathway enrichment including the biological process (BP), molecular function (MF), cellular component (CC), and KEGG pathway of the DEGs. Moreover, we performed two modules and confirmed their enriched pathways. The core genes out of the 15 hub ones were found, and the interactions between any of them were detected with the help of GEPIA. After the analysis of the core status and biological function of any hub gene, we performed flow cytometry (FCM) to count mononuclear cells, confirming the findings.

## 2. Results

### 2.1. Identification of DEGs and Hub Genes

**Figure 1:**
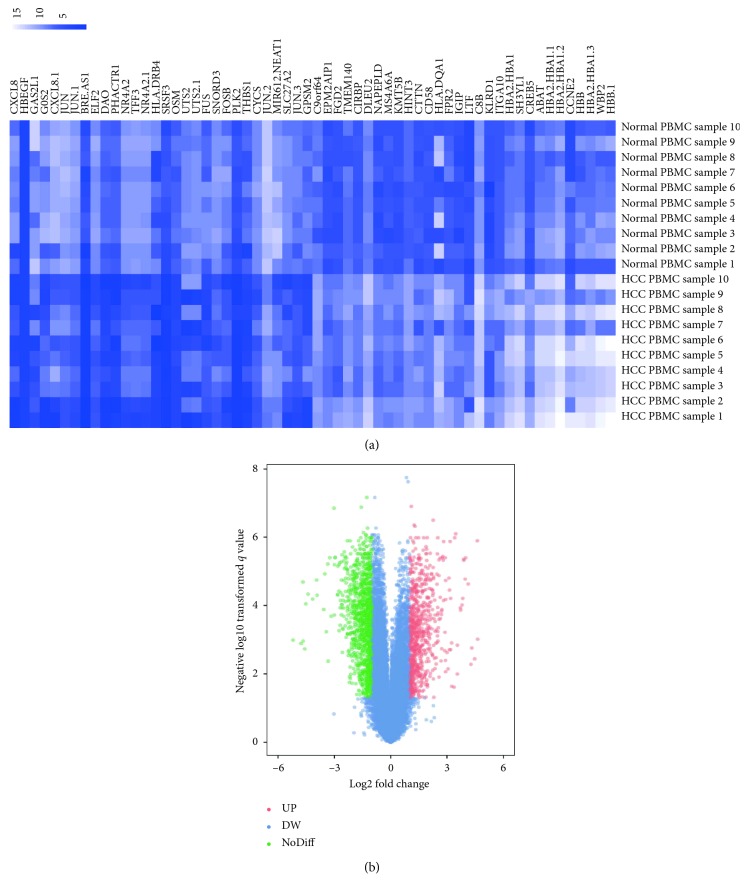
Identification of DEGs of PBMC in GSE49515. (a) Heatmap of the expression levels of the top 50 DEGs employed the GEO2R online analysis tool based on *P* value < 0.05 and logFC ≤ −2 or logFC ≥ 2 criteria. (b) Volcano plot about total 344 DEGs from GSE49515. The red dots represent 188 upregulated DEGs, while the green dots represent 156 downregulated DEGs. The blue dots denote the no-differentially expressed genes.

A comparison of 10 HCC samples with 10 normal samples in our study was performed by employing the GEO2R online analysis tool based on *P* value < 0.05 and logFC ≤ −2 or logFC ≥ 2 criteria. A total of 344 DEGs were picked up after analyzing GSE49515, 188 of which were upregulated while 156 were downregulated (Figure 1(b)). The expression levels of the top 50 DEGs were displayed in a heat map to visualize the results (Figure 1(a)).

### 2.2. GO Function- and KEGG Pathway-Enrichment Analysis

To gain a more extensive and in-depth knowledge of those selected DEGs, we use DAVID to analyze significantly enriched GO function and KEGG pathways. After inputting all of the DEGs to DAVID online analysis tool, we obtained the GO analysis of these upregulated DEGs and downregulated DEGs. The results showed that these DEGs were mainly enriched in biological processes (BP), including apoptotic process, immune response, and inflammatory response, among which were positive regulation of NF-*κ*B transcription factor activity and cell-cell signaling for downregulation, positive regulation of angiogenesis, negative regulation of cell proliferation, positive regulation of cell proliferation, mitotic spindle organization, and neutrophil chemotaxis for upregulation. Concerning molecular function (MF), the downregulated DEGs were particularly related to receptor binding, iron ion binding, haptoglobin binding, oxygen transporter activity, and peroxidase activity, while the upregulated DEGs were mainly implicated with nucleotide binding and ubiquitin protein ligase binding. Besides, GO cell component (CC) analysis indicated that the downregulated DEGs were mainly enriched in cytosol, extracellular exosome, Golgi membrane, blood microparticle, and nuclear chromosome (telomeric region) and the upregulated DEGs were principally enriched in nucleus, nucleoplasm, platelet alpha granule and extracellular space (Table 1).

Afterwards, we analyzed the most significantly enriched KEGG pathway of the upregulated and downregulated DEGs, which is shown in Table 2. The downregulated DEGs were involved in measles, influenza A, rheumatoid arthritis, antigen processing and presentation, and legionellosis, while the upregulated DEGs were involved in bladder cancer, rheumatoid arthritis, malaria, herpes simplex infection, and osteoclast differentiation. The scatter plots in [Supplementary-material supplementary-material-1] (A, B, and C) show a GO and KEGG pathway-enrichment plot of HCC.

### 2.3. Hub Genes and Module Screening from PPI Network

**Figure 2:**
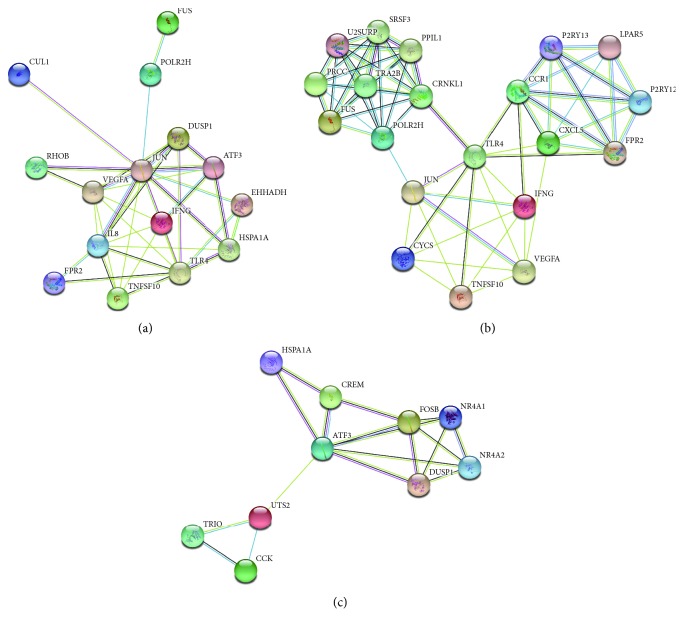
PPI network and module analysis of HCC. (a) PPI network of the top 15 hub genes via the information of the STRING protein query with maximum number of interactors ≤ 5 and confidence score ≥ 0.4. (b) PPI network of Module 1. (c) PPI network of Module 2.

Besides, 15 hub genes from Cytoscape software were identified in accordance with a high degree of connectivity selected (Table 3). We built the PPI network of the top 15 hub genes via the information of the STRING protein query from public databases (Figure 2(a)). The top 15 hub genes with a higher degree of connectivity are as follows: JUN, IL8, VEGFA, TLR4, IFNG, TNFSF10, EHHADH, ATF3, FUS, DUSP1, HSPA1A, CUL1, FPR2, POLR2H, and RHOB. Based on the GO function and KEGG pathway analysis of the profile, we uncovered JUN, VEGFA, TNFSF10, and TLR4 that were enriched in immune response-related pathway.

Moreover, we used MCODE plug-in to detect the highest modules in the PPI network. We chose the top 2 modules, and GO function- and KEGG pathway-enrichment analysis indicated that Module 1 was related to immune response, apoptotic process, and epithelial cell migration, while Module 2 was associated with a signaling pathway and cellular response to various substances (Figures 2(b) and 2(c), Table 4).

### 2.4. The Kaplan–Meier Plotter and Expression Level of Hub Genes

**Figure 3:**
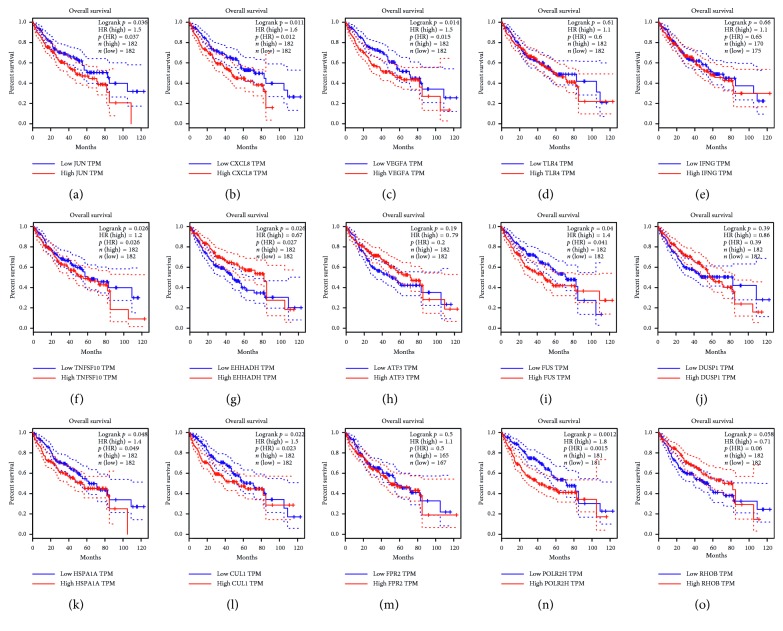
Survival curve of HCC patients of 15 hub genes. Prognostic value of 15 genes (JUN, IL8 (called as CXCL8), VEGFA, TLR4, TNFSF10, IFNG, EHHADH, ATF3, FUS, DUSP1, HSPA1 A, CUL1, FPR2, POLR2H, and RHOB) in HCC. *P* < 0.05 was regarded statistically different.

The prognosis of the top 15 hub genes was analyzed in http://gepia.cancer-pku.cn/. We found that the expression of JUN (*P*=0.037) was related to worse overall survival (OS) for HCC patients, as well as IL8 (called as CXCL8) (*P*=0.011), VEGFA (*P*=0.014), EHHADH (*P*=0.026), FUS (*P*=0.04), HSPA1A (*P*=0.048), CUL1 (*P*=0.022), and POLR2H (*P*=0.0012). The level of TLR4 (*P*=0.61), IFNG (*P*=0.66), TNFSF10 (*P*=0.26), ATF3 (*P*=0.19), DUSP1 (*P*=0.39), FPR2 (*P*=0.5), and RHOB (*P*=0.058) had no obvious difference in overall survival of HCC patients (Figure 3). However, the survival curves are analyzed with liver tissue, which can only indirectly explain the importance of hub genes in PBMC. These hub genes in PBMC which can be the biomarkers for early diagnosis may not be easy to detect in liver tissue.

**Figure 4:**
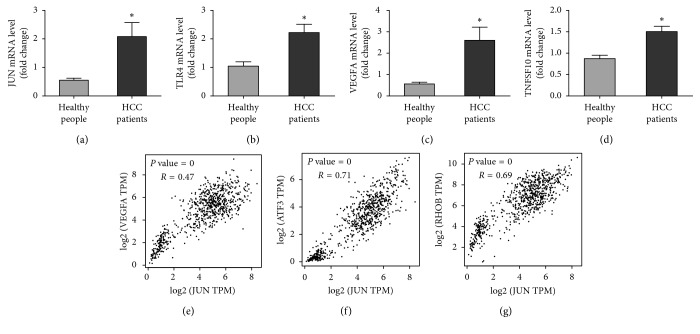
The mRNA level of hub genes and correlation analysis of any two hub genes in GEPIA. (a–d) The mRNA level of 4 hub genes (JUN, TLR4, VEGFA, and TNFSF10) in PBMC (^*∗*^Differences between the groups were significant (*P* < 0.05)). (e) JUN and VEGFA correlation analysis (*P* value = 0, *R*=0.47). (f) JUN and ATF3 correlation analysis (*P* value = 0, *R*=0.71). (g) JUN and RHOB correlation analysis (*P* value = 0, *R*=0.69).

Then, we employed DAVID to analyze the correlation of 15 hub genes. We found JUN, IFNG, VEGFA, TLR4, and TNFSF10 are the 5 high-degree-of-connectivity genes which are fully associated with immune response, inflammatory response, and HIF-1 signaling pathway (Table 5). Then, in order to confirm the most relevant hub genes, we used correlation analysis in GEPIA, and we detected JUN and VEGFA, JUN and ATF3, and JUN and RHOB are distinctly correlated (*P* value = 0, *R*=0.47; *P* value = 0, *R*=0.71; *P* value = 0, *R*=0.69), which means JUN may be the core gene of HCC (Figures 4(e)–4(g)).

### 2.5. Gene Set Enrichment Analysis

**Figure 5:**
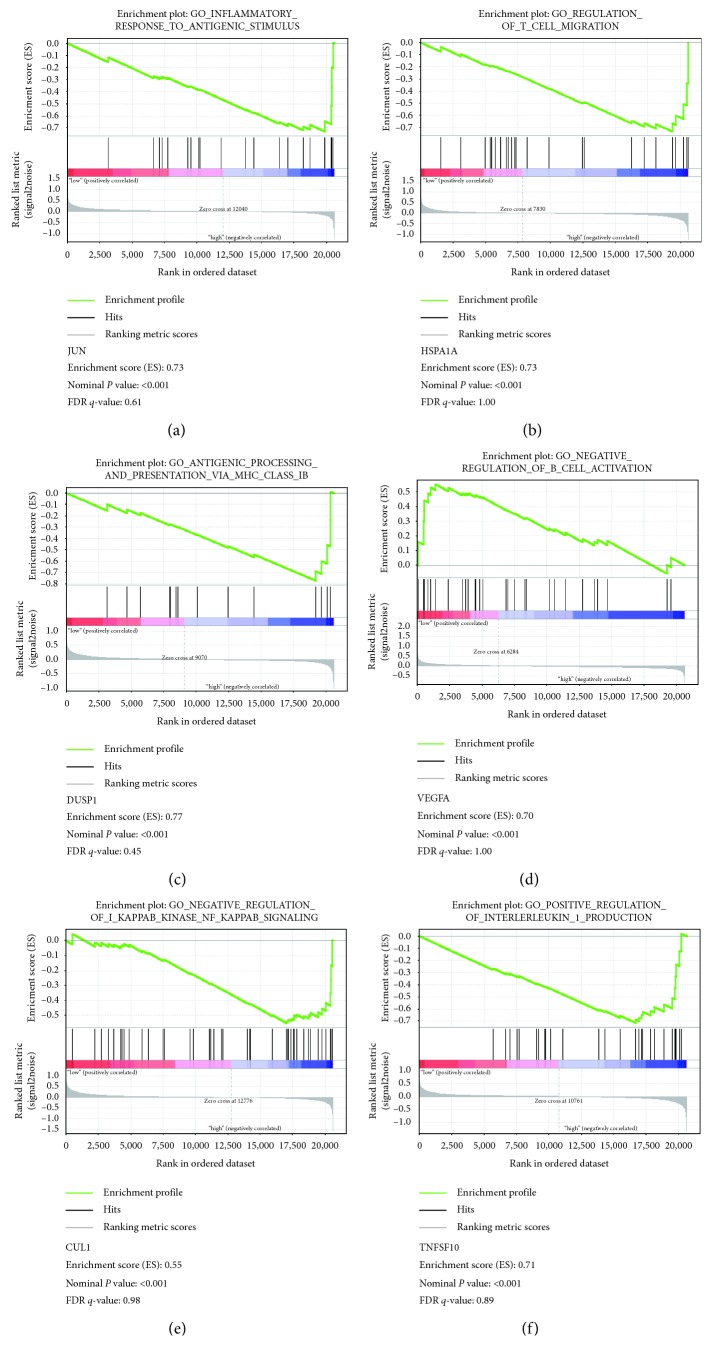
Gene set enrichment analysis of HCC. (a) JUN is negatively correlated with inflammatory response to antigenic stimulus (b) HSPA1A is negatively correlated with regulation of T cell migration. (c) DUSP1 is negatively correlated with antigen processing and presentation via MHC class IB. (d) VEGFA is positively correlated with negative regulation of B cell activation. (e) CUL1 is negatively correlated with negative regulation of NF-kB signaling. (f) TNFSF10 is negatively correlated with positive regulation of IL-1 production.

In order to make the further function of the hub gene clear, GSEA was used to map into GO analysis and KEGG pathways database. Under the cutoff criteria nominal *P* value < 0.05, │enrichment score (ES)│ > 0.6, and gene size ≥ 100, six functional gene sets were enriched in total, which were particularly centralized on pathway associated with immune response and inflammatory response. Six pathways were “inflammatory response to antigenic stimulus,” “regulation of T cell migration,” “antigen processing and presentation via MHC class IB,” “negative regulation of B cell activation,” “negative regulation of NF-kB signaling,” and “positive regulation of interleukin-1 production” (Figure 5).

### 2.6. Identification of Biomarkers

To confirm the results we have stated above, we test the mRNA level of four key genes we predicted (JUN, TLR4, VEGFA, and TNFSF10). We found the mRNA level of JUN, TLR4, VEGFA, and TNFSF10 in the PBMC of HCC patients was significantly upregulated in the PBMC of HCC patients (*P* < 0.05), of which VEGFA increased obviously (*P* < 0.01) (Figures 4(a)–4(d)).

### 2.7. Immune Subversion in HCC

**Figure 6:**
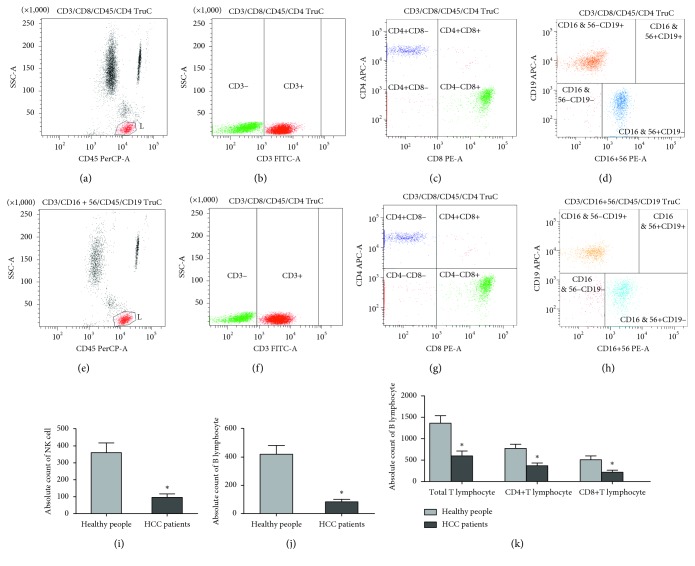
Assessment lymphocyte subset by FCM from 20 patients with HCC and 20 healthy individuals. (a, e) Identification of total lymphocyte (CD45+) cell count in healthy individual and HCC patient blood. (b, f) Identification of total T lymphocyte (CD3+) cell count in healthy individuals' and HCC patients' blood. (c, g) Different expression of CD4+ T lymphocyte (CD4+ CD8−) and CD8+ T lymphocyte (CD4− CD8+) in healthy individuals' and HCC patients' blood, respectively. (d, h) Identification of B lymphocyte (CD16 & 56− CD19+) cell count and NK cell count (CD16 & 56+ CD19−) in healthy individuals' and HCC patients' blood, respectively. (i) Absolute count of NK cell in healthy individuals' and HCC patients' blood, respectively. (j) Absolute count of B lymphocyte in healthy individuals' and HCC patients' blood, respectively. (k) Absolute count of various T lymphocyte in healthy individuals' and HCC patients' blood. (^*∗*^Differences between the groups were significant (*P* < 0.05)).

According to our prediction, the negative regulation of immune cells in HCC patients' peripheral blood significantly occurred detected by FCM (Figures 6(a)–6(h)). In detail, compared with the healthy subjects, the levels of T lymphocytes in peripheral blood, both helper T cells and cytotxic T cells, were significantly lower in HCC patients, which is mainly the immune mechanism of tumor patients (*P* < 0.05) (Figure 6(k)). Meanwhile, the B lymphocytes as well as NK cells also decreased in HCC patients, especially NK cells (*P* < 0.05) (Figures 6(i) and 6(j)). Taken together, our experiment of FCM demonstrated that T cell migration and B cell activation in adaptive immune and NK cells inhibition in innate immune were important mechanisms and could do further research in future.

## 3. Discussion

In recent decades, the morbidity and mortality of HCC have been increasing worldwide. Although the early diagnosis and treatment have developed a lot recently, the overall survival rates of HCC is still poor [16]. Therefore, the sensitive and specific biomarkers for HCC are urgently necessary. High-throughput studies can develop the thorough exploration of the vital mechanisms which lead to HCC. In our study, we identified DEGs between 10 HCC samples and 10 normal samples from the GEO database of GSE49515. In order to increase the statistical power of these DEGs, we defined that the absolute value of the logarithm (base 2) fold change (logFC) greater than 2 and a total of 344 DEGs were captured, including 188 upregulated genes and 156 downregulated genes. In order to have an in-depth detection of these DEGs, we employed GO function, KEGG pathway, PPI network, and connectivity analysis of these DEGs, via which we found that HCC-related genes and pathways have great importance in cancer initiation and progression.

There were plenty of mechanisms uncovered to contribute to the development of HCC, but the predominant mechanism implicated with tumorigenesis is still controversial, thereby causing difficulties to the diagnosis and treatment of HCC. GO term enrichment and PPI analysis in our study disclosed that downregulated DEGs were mainly associated with immune response. Our experimental results detecting the mononuclear cells in peripheral blood mononuclear cells of HCC patients and healthy individuals further confirmed the important role of immune subversion.

Immune response is well recognized to play a vital part in the initiation and progression of carcinogenesis because the development of HCC obviously records failure of tumor immune control which stands for immune subversion by the tumor environment [2]. To our knowledge, protective immune surveillance of tumor is mainly conducted by tumor-directed NK cells and lymphocytes, which can effectively identify and eradicate malignant cells [6]. On the one hand, for the reason that innate immune cells are capable of eliminating malignant cells and pertaining to the first-line defense to restrain tumor initiation and progression [17], they act as an essential player in the immunological surveillance. On the other hand, adaptive immune cells including B lymphocytes and T lymphocytes develop along with innate immune responses, which finally target tumor-associated antigens (TAAs) [18]. To be more specific, once the immune cells in peripheral blood are unable to generate or be recruited, specific immunity and nonspecific immunity function will degrade, eventually causing the rapid growth of carcinoma tissue. The quantity of immune cells is associated with many immune-related genes which may be differentially expressed during tumor manifestation and progression.

NK cells (CD3−/CD56+) reside in the liver and account for about 30% to 50% of the hepatic lymphocytes in humans [17]. Therefore, NK cells constitute a corresponding effector cell population of innate immune cells contributing to tumor surveillance within the liver. In our data from FCM, we identified that NK cells in HCC patients' peripheral blood were significantly lower than those in the blood from normal people. Additionally, our GO analysis indicated that the downregulated DEGs were mainly related to immune response including immune cells manifestation and recruitment and release of cytokines. We found that there were 4 genes (JUN, VEGFA, TNFSF10, and TLR4) closely related to the downregulation of immune response which occurs in HCC. Mgrditchian et al. [19] found that the phosphorylation of JUN could induce NK cell infiltration into the tumor bed by inducing the transcription of CCL5, which eventually results in targeted autophagy. However, NK cells produce vascular endothelial growth factor A (VEGFA) in tumor tissues, which may enhance the formation of tumor by the way of angiogenesis. The presence of VEGFA-secreting NK cells is associated with a poor prognosis and depends on the type and stage of the tumor [20]. In our GO analysis, we detected that the upregulated DEGs were particularly associated with the positive regulation of angiogenesis, which indirectly verified that VEGFA-secreting NK cells might play an important part in the HCC. Wagner et al. reported that membrane-bound tumor necrosis factor ligand superfamily member 10 (TNFSF10) on the NK cells can supplement the perforin/granzyme pathway in a NK cell-mediated cytotoxicity, which can enhance the NK cell function of tumor elimination [21]. Besides, there were a few reports which showed that some antitumor substances can induce NK cells to proliferate and release IFN-*γ* via TLR4; thus, we predicted that TLR4 might be an important target to influence HCC development [22–24]. Our result suggested that innate immune cells, especially the function of NK cells, are downregulated via some hub genes in HCC.

Adaptive immunity including humoral immunity and cellular immunity is highly specific elimination of transformed cells, which protects from tumor manifestation and progression. B lymphocytes, CD4+ T lymphocytes, and CD8+ T lymphocytes are vital members in adaptive immunity [25]. Equally, we employed FCM to detect the B lymphocytes and two T lymphocyte subsets which verified the reduction of adaptive immune function in HCC. We predicted the mechanisms of B lymphocytes and T lymphocyte declining may also be associated with the four hub genes (JUN, VEGFA, TNFSF10, and TLR4) via GO and KEGG analysis. c-JUN NH_2_-terminal kinase (JNK) signaling pathway was implicated in various T cell functions. The JNKs are synergistically activated by stimulation of the TCR with antibodies to its CD3 component and the CD28 auxiliary receptor, which was related to T cell activation [26]. Patterson et al. [27] discovered that Ig*α* non-ITAM tyrosine 204 promoted T-independent B cell proliferation and differentiation via phosphorylation of JUN. In addition, tumor can produce VEGFA, which increases expression of inhibitory immune checkpoints mediating T cell exhaustion on intratumoral CD8+ T cells [28]. The previous report identified that VEGFA directly triggers regulatory T cells (Treg) proliferation resulting in tumors escape, which can become a therapeutic goal in the future [29]. TNFSF10 influences T cells function, which cannot only enhance the maximal suppressive function of Treg cells leading to the antitumor effect but also be involved in T cell-mediated killing of cancer cells [30, 31]. Furthermore, Fang et al. demonstrated that TLR4 appears to induce antitumor T cell response by activating dendritic cells [32]. Similarly, there is research confirming that TLR4 can promote tumor-specific cytotoxic T cell responses [33]. Taken together, from our perspective based on the GO and KEGG analyses and FCM, the regulation of immune-related genes including JUN, VEGFA, TNFSF10, and TLR4 can influence the immune response and be involved in the occurrence and progression of HCC. Drugs targeting immune response of peripheral mononuclear cells may be the potential candidates for therapy of HCC.

The PPI network could form a visible framework for a better understanding of the function of the proteome [34]. From the enriched pathways of top 2 modules, we discovered that the interactions among the proteins in HCC are particularly associated with pathways of immune response and cytokine-cytokine receptor interaction. It emphasizes that immune-related gene interaction can regulate the tumor-associated immune surveillance. Therefore, we utilized DAVID to uncover the correlation of 15 hub genes. Coincidentally, the 4 genes, JUN, VEGFA, TNFSF10, and TLR4, have the high degree of connectivity, especially JUN and VEGFA. We predicted that JUN can regulate mononuclear cells to release VEGFA, which may promote tumor angiogenesis, which was proved as the reason for tumor initiation and progression. Furthermore, we analyzed the gene set enrichment to make sure of the primary function of the 4 hub genes. We found these 4 hub genes were inextricably linked with the various processes of immune and inflammatory responses like the regulation of T and B cell migration and antigen processing via MHC class I B. Hence, monitoring the immune process of tumor immunity including immune-related genes and cytokines is of great importance for the diagnosis and treatment of HCC.

Moreover, HCC patients always used to suffer from hepatitis infected by hepatitis B virus (HBV) and hepatitis C virus (HCV). Patients who suffer cirrhosis or HCC from chronic virus hepatitis account for 90% [35]. It is consistent with our GO and KEGG analyses that the inflammation is an important process of HCC. JUN, VEGFA, TNFSF10, and TLR4 are connectivity genes involved in the inflammatory response. For instance, there was a report that stated JUN can act as an intermediate in antiviral immunity and TLR4 recognizes bacterial ligands to constrain the ability of antiviral immunity which combine bacterial and viral infections [36, 37]. As a result, we hypothesized that JUN, VEGFA, TNFSF10, and TLR4 in PBMC can also regulate virus infection of HCC patients. Detecting these genes can early protect patients from HCC occurrence and development as well as treat them early.

In conclusion, we provide a comprehensive and novel analysis of gene expression profiles to recognize DEGs which may play a core part in the development and prognosis in patients with HCC. Genes implicated with immune and inflammatory response were significantly altered in HCC patients. In order to acquire more precise correlation results, we plan to carry out subsequent authentication experiment later to prove these predictive results. Taken together, we found that JUN, VEGFA, TNFSF10, and TLR4 in PBMC play core roles in the immune response of HCC. Hence, these 4 genes may serve as potential serum biomarkers combining with AFP and targets of immunotherapy. We expect this analysis method will offer accurate and valuable information for future study on the molecular mechanisms of HCC and supply evidences for the detection of new diagnosis biomarkers and therapeutic strategies.

## 4. Materials and Methods

### 4.1. Microarray Data

We downloaded the gene expression profile of GSE49515 from GEO database. We chose a total of 20 samples, involving 10 cases of HCC and 10 healthy cases of purified RNA of peripheral blood mononuclear cells (PBMCs) in GSE49515, which was based on Agilent Gpl570 platform ([HG-U133_Plus_2] Affymetrix Human Genome U133 Plus 2.0 Array) by Shi et al. We also get the Series Matrix File of GSE49515 from GEO database.

### 4.2. Screen Genes of Differential Expression

The analysis of DEGs between HCC samples and normal liver tissue samples was performed by using GEO2R (https://www.ncbi.nlm.nih.gov/geo/geo2r/), an online analysis method for GEO database on the basis of R language. We regulated DEGs as differentially expressed with logFC < −2 (upregulated genes) or logFC > 2 (downregulated genes), according to the criteria described in [38, 39]. The adjusted *P* value < 0.05 was treated as statistically significant in order to reduce the false-positive rate. Thereafter, 344 DEGs were picked up, containing 188 upregulated genes and 156 downregulated genes. We utilized visual hierarchical cluster analysis to get the heatmap and volcano plot of two groups by ImageGP (http://www.ehbio.com/ImageGP/index.php/Home/Imdex/index.html) after the correlative raw data of TXT files were downloaded.

### 4.3. Gene Ontology and KEGG Pathway Analysis of DEGs

Gene ontology (GO) analysis is a common framework which can annotate genes and gene products including functions of cellular components, biological pathways, and molecular function [40]. Kyoto Encyclopedia of Genes and Genomes (KEGG) contains a set of genomes and biological pathways related to disease and drugs online database, which essentially is a resource for systematic understanding of biological system and certain high-level genome functional information [41]. The Database for Annotation, Visualization and Integrated Discovery (DAVID, http://david.ncifcrf.gov) is an online bioinformatics database. It has covered many biological data and relevant analysis tools, and then provided tools for the biological function annotation information for plenty of genes or proteins [42]. *P* < 0.05 was considered as the cutoff criterion with significant difference. We could visualize the key biological processes, molecular functions, and cellular components and pathways of DEGs by using DAVID online database. And the scatter plot was performed by ImageGP according to the results of GO and KEGG pathway.

### 4.4. PPI Network and Module Analysis

Search Tool for the Retrieval of Interacting Genes (STRING) is an online tool for the assessment and integration of the protein-protein interaction (PPI) information, containing physical and functional associations. It covered 9,643,763 proteins from 2031 organisms in STRING version 10.0 [43]. We drew DEGs using STRING to evaluate the interactional associations among them, thereby utilizing the Cytoscape software to build a PPI network. In the meantime, we set maximum number of interactors = 0, confidence score ≥0.4 as the cutoff criterion. Moreover, the Molecular Complex Detection (MCODE) app was utilized to screen modules of the PPI network in Cytoscape in line with degree cutoff = 2, *k*-core = 2, node score cutoff = 0.2, and max. depth = 100. And we chose the top 15 genes with a high degree of connectivity as hub genes. The pathway analysis of genes in every module was worked out according to DAVID. Then, 15 hub genes were also mapped into STRING with maximum number of interactors ≤5 and confidence score ≥0.4. GO and KEGG pathway analyses were also used to explain the potential information.

### 4.5. Comparison of the Hub Genes Expression Level

GEPIA (http://gepia.cancer-pku.cn/index.html) is a newly developed interactive web server which analyzes the RNA-sequencing expression data of 9,736 tumors and 8,587 normal samples from the TCGA and the GTEx projects, employing a standard processing pipeline. GEPIA provides customizable functions such as tumor and normal differential expression analysis, profiling according to pathological stage or cancer types, patient survival analysis, similar gene detection, correlation analysis, and dimensionality reduction analysis [44]. In our study, we mainly used the correlation to reveal the relevance out of any two hub genes in HCC and normal people's peripheral blood. Moreover, two suspicious genes that demonstrated a good manner in the scatter diagram were chosen.

### 4.6. Survival Analysis of Hub Genes

We used GEPIA database to analyze the relapse-free and overall survival information related to the hub genes. The hazard ratio (HR) with 95% confidence intervals and log rank *P* value were computed and showed on the plot. *P* < 0.05 was statistically significant.

### 4.7. Gene Set Enrichment Analysis (GSEA)

We divided 20 HCC samples from GSE49515 into two groups (high and low) on the basis of expression level of several key genes, and median expression value was regarded as the cutoff point. In order to explain the potential function of these key genes, GSEA (http://software.broadinstitute.org/gsea/index.jsp) was operated between the two groups. Annotated genes were selected as the reference gene sets. Nominal *P* value < 0.05, gene size ≥ 100, and│enrichment score (ES)│ > 0.5 were regarded as the cutoff criteria.

### 4.8. Identification of Biomarkers

Based on the information in the individual MCODE modules, the node with the highest score was selected as the hub gene in GSE49515. According to our analysis of KEGG and PPI, we confirm four key genes (JUN, TLR4, VEGFA, and TNFSF10) which can be the noninvasive biomarkers in early HCC. To verify the vital role of the four key genes in HCC, the mRNA level of these four genes were measured in the PBMC of 20 HCC patients and 20 healthy individuals. Primers used for PCR are listed as follows: JUN-F: ACAGAGCATGACCCTGAACCT; JUN-R: TGTGCCCGTTGCTGGAC; TLR4-F: ATCAAGGACCAGAGGC; TLR4-R: CACTGAGGACCGACAC; VEGFA-F: GACGGACAGACAGACAGACACC; VEGFA-R: GAAGCGAGAACAGCCCAGA; TNFSF10-F: AGTGGCATTGCTTGTTT; TNFSF10-R: GAGCTGACGGAGTTGC.

### 4.9. Reidentification of Immune Subversion in HCC

Meantime, in order to confirm the key role of immune subversion in HCC, we screened peripheral blood mononuclear cells (PBMCs) using flow cytometry (FCM), which were obtain from 20 patients with HCC and 20 healthy individuals (ethical approval for the study was obtained from the Zhongnan Hospital of Wuhan University (no. 2019016)). Thereafter, we detected the level of some typical immune cells in PBMCs of HCC and healthy people via the molecules on the immune cell surface using BD FACSCanto II. The samples we detected were from anticoagulated blood with BD Multi TEST IMK Kit (Catalog No. 340503). The total level of T-lymphocytes was screened through CD3 molecules which includes helper lymphocyte T subsets detected by CD3 and CD4 molecules and inhibitory T lymphocyte subsets detected by CD3 and CD8 molecules. B lymphocytes and natural killer (NK) cells were determined by CD19, CD16, and CD56 molecules, respectively. This study was carried out according to legal requirements and supported by the Ethics Committee of the Zhongnan Hospital of Wuhan University. The HCC patients we chose are stage 0 and A of the Barcelona Clinic Liver Cancer (BCLC) stage as well as T1 and T2 of the TNM stage. It means the HCC patients are in the early stage of HCC. Besides, all patients did not take any invasive therapies, like tumor excision, interventional therapy, and hepatic artery embolism. The clinical characteristics of the participants we choose are summarized in Table 6.

### 4.10. Statistical Analysis

All values were reported as means ± SD. Statistical significance was analyzed by SPSS 19.0 software. Differences were considered significant when *P* < 0.05.

## Figures and Tables

**Table 1:** Gene ontology analysis of differentially expressed genes associated with hepatocellular carcinoma.

	Category	Term	Count	%	*P* value
Upregulated	GOTERM_BP_DIRECT	GO:0045766∼positive regulation of angiogenesis	5	<0.001	<0.001
	GOTERM_BP_DIRECT	GO:0008285∼negative regulation of cell proliferation	5	0.01	0.01
	GOTERM_BP_DIRECT	GO:0008284∼positive regulation of cell proliferation	5	0.02	0.02
	GOTERM_BP_DIRECT	GO:0007052∼mitotic spindle organization	3	<0.001	<0.001
	GOTERM_BP_DIRECT	GO:0030593∼neutrophil chemotaxis	3	0.02	0.02
	GOTERM_CC_DIRECT	GO:0005634∼nucleus	20	0.12	0.01
	GOTERM_CC_DIRECT	GO:0005654∼nucleoplasm	11	0.07	0.02
	GOTERM_CC_DIRECT	GO:0031091∼platelet alpha granule	2	0.01	0.04
	GOTERM_CC_DIRECT	GO:0005615∼extracellular space	9	0.06	0.05
	GOTERM_MF_DIRECT	GO:0000166∼nucleotide binding	5	0.03	0.04
	GOTERM_MF_DIRECT	GO:0031625∼ubiquitin protein ligase binding	3	0.02	0.05

Downregulated	GOTERM_BP_DIRECT	GO:0006915∼apoptotic process	9	0.06	0.02
	GOTERM_BP_DIRECT	GO:0006955∼immune response	8	0.05	0.02
	GOTERM_BP_DIRECT	GO:0006954∼inflammatory response	7	0.04	0.03
	GOTERM_BP_DIRECT	GO:0007267∼cell-cell signaling	6	0.04	0.05
	GOTERM_BP_DIRECT	GO:0051092∼positive regulation of NF-kappaB transcription factor activity	6	0.04	<0.001
	GOTERM_CC_DIRECT	GO:0005829∼cytosol	33	0.21	0.01
	GOTERM_CC_DIRECT	GO:0070062∼extracellular exosome	26	0.16	0.04
	GOTERM_CC_DIRECT	GO:0000139∼golgi membrane	9	0.06	0.03
	GOTERM_CC_DIRECT	GO:0072562∼blood microparticle	6	0.04	<0.001
	GOTERM_CC_DIRECT	GO:0000784∼nuclear chromosome, telomeric region	4	0.03	0.05
	GOTERM_MF_DIRECT	GO:0005102∼receptor binding	7	0.04	0.03
	GOTERM_MF_DIRECT	GO:0005506∼iron ion binding	5	0.03	0.02
	GOTERM_MF_DIRECT	GO:0031720∼haptoglobin binding	3	0.02	<0.001
	GOTERM_MF_DIRECT	GO:0005344∼oxygen transporter activity	3	0.02	<0.001
	GOTERM_MF_DIRECT	GO:0004601∼peroxidase activity	3	0.02	0.01

**Table 2:** KEGG pathway analysis of differentially expressed genes associated with hepatocellular carcinoma.

Category	Term	Count	%	*P* value	Genes
Up regulated	ssc05219: bladder cancer	4	0.02	0.002	VEGFA, CXCL8, HBEGF, THBS1
	ssc05323: rheumatoid arthritis	5	0.03	0.002	JUN, IFNG, VEGFA, CXCL8, ITGB2
	ssc05144: malaria	4	0.02	0.004	IFNG, CXCL8, ITGB2, THBS1
	ssc05168: herpes simplex infection	5	0.03	0.024	SRSF3, JUN, IFNG, CYCS, CUL1
	ssc04380: osteoclast differentiation	4	0.02	0.047	FOSL2, SQSTM1, JUN, IFNG

Down regulated	hsa05162: measles	8	0.05	<0.001	CCNE2, TNFSF10, EIF2S1, TLR4, HSPA1A, HSPA1B, MSN, TLR7
	hsa05164: influenza A	8	0.05	0.001	TNFSF10, EIF2S1, TLR4, HSPA1A, HSPA1B, TLR7, HLA-DQA1, HLA-DRA
	hsa05323: rheumatoid arthritis	6	0.04	0.001	CXCL5, TLR4, LTB, HLA-DQA1, HLA-DRA, IL11
	hsa04612: antigen processing and presentation	5	0.03	0.004	HSPA1A, HSPA1B, KLRD1, HLA-DQA1, HLA-DRA
	hsa05134: legionellosis	4	0.03	0.012	NLRC4, TLR4, HSPA1A, HSPA1B

**Table 3:** Top 15 hub genes with higher degree of connectivity.

Name	Degree	*P* value	Log FC
JUN	36	1.25E-06	2.933038
CXCL8	26	5.79E-05	4.628443
VEGFA	23	4.12E-07	2.049058
TLR4	18	2.46E-04	−2.256283
IFNG	15	1.63E-04	2.882006
TNFSF10	14	9.28E-05	−2.08173
EHHADH	13	2.13E-05	−2.241576
ATF3	12	1.89E-05	2.081481
FUS	12	8.17E-05	3.243027
DUSP1	11	5.95E-06	2.36054
HSPA1A	11	1.67E-07	−2.00933
CUL1	10	2.24E-05	2.195652
FPR2	10	6.73E-06	−3.173126
POLR2H	10	2.34E-07	−2.370116
RHOB	10	1.14E-05	2.368394

**Table 4:** The enriched pathway of top 2 modules.

Module	Term	*P* value	FDR	Genes
Module 1	Immune response	0.02	22.28	TNFSF10, JUN, CCR1
	Positive regulation of cysteine-type endopeptidase activity involved in apoptotic process	0.02	24.07	TNFSF10, CYCS
	Cytokine-cytokine receptor interaction	0.01	14.95	TNFSF10, CCR1, IFNG, VEGFA
	Positive regulation of epithelial cell migration	0.02	21.94	JUN, IFNG
	HIF-1 signaling pathway	0.02	22.98	IFNG, VEGFA, TLR4

Module 2	Cellular response to corticotropin-releasing hormone stimulus	<0.001	1.74	NR4A2, NR4A1
	Intracellular receptor signaling pathway	<0.001	6.26	NR4A2, NR4A1
	Steroid hormone-mediated signaling pathway	0.01	23.88	NR4A2, NR4A1
	MAPK signaling pathway	<0.001	0.90	DUSP1, NR4A1, HSPA1A
	Negative regulation of cysteine-type endopeptidase activity involved in apoptotic process	0.02	22.82	NR4A1, HSPA1A

**Table 5:** KEGG pathway analysis of top 15 hub genes with higher degree of connectivity.

Term	Count	%	*P* value	Genes	FDR
ssc05323: rheumatoid arthritis	4	0.18	1.41E-04	JUN, IFNG, VEGFA, TLR4	0.14
ssc05164: influenza A	4	0.18	9.95E-04	JUN, IFNG, TNFSF10, TLR4	1.04
ssc05321: inflammatory bowel disease (IBD)	3	0.13	0.002	JUN, IFNG, TLR4	2.66
ssc04066: HIF-1 signaling pathway	3	0.13	0.007	IFNG, VEGFA, TLR4	7.01
ssc04060: cytokine-cytokine receptor interaction	3	0.13	0.031	IFNG, VEGFA, TNFSF10	27.91

**Table 6:** Clinical characteristics of participants.

Group	Hepatocellular carcinoma (HCC) (*n* = 20)	Healthy individuals
Gender		
Male	13	12
Female	7	8
Age	55 (42–76)	43 (20–65)
Alpha-fetoprotein (AFP)		
>20 ng/ml	20	0
<20 ng/ml	0	20
Tumor size	5.57 (2.7–8.6)	—
≤3 cm	2	—
3–5 cm	9	—
>5 cm	7	—
Not available	2	—
Barcelona Clinic Liver Cancer (BCLC) stage		
Stage 0	0	—
Stage A	9	—
Stage B	7	—
Stage C	0	—
Stage D	0	—
Not available	4	—
Viral infection		
HBV	20	0
HCV	0	0
Alcohol	0	0
None	0	20
TNM stage		
T1	18	—
T2	2	—
T3	0	—
T4	0	—
Not available	0	—

## Data Availability

The figure and table data used to support the findings of this study are included within the article.

## Supplementary Materials

Supplementary Materials Supplement 1: the scatter plots of GO function- and KEGG pathway-enrichment plot of HCC. (A) and (B) GO-pathway enrichment scatter plot of HCC. (C) KEGG pathway-enrichment scatter plots of HCC.
