# Supplementary figures and images for: Noninvasive Identification of Immune-Related Biomarkers in Hepatocellular Carcinoma
Source: J Oncol. 2019 Aug 18;2019:2531932. doi: 10.1155/2019/2531932 (PMC6721356; doi:10.1155/2019/2531932)

**Supplement 1**


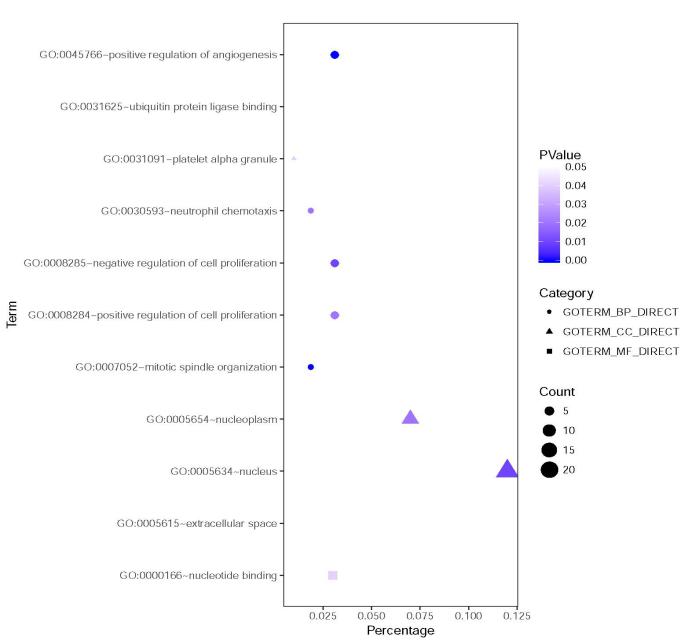

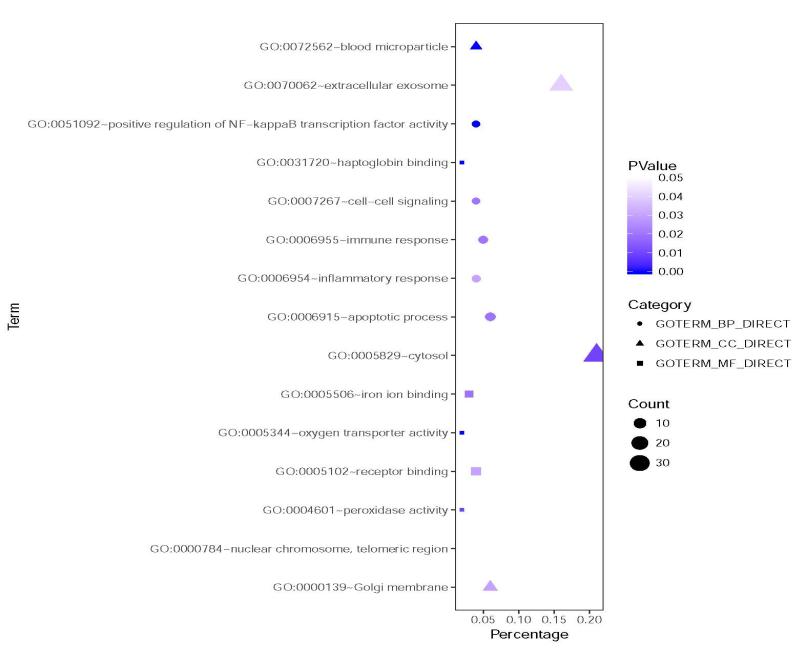

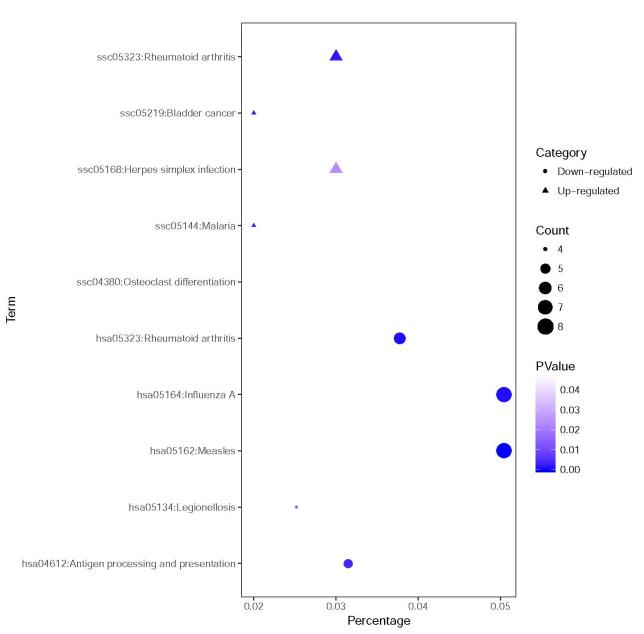


A

B

C

Supplement: Supplementary Materials — Supplement 1: the scatter plots of GO function- and KEGG pathway-enrichment plot of HCC. (A) and (B) GO-pathway enrichment scatter plot of HCC. (C) KEGG pathway-enrichment scatter plots of HCC. [file 2531932.f1.docx]
